# Secrecy Capacity Enhancement in Active IRS-Assisted UAV Communication System

**DOI:** 10.3390/s23094377

**Published:** 2023-04-28

**Authors:** Jiansong Miao, Tongjie Li, Shanling Bai, Shi Yan, Yan Zhao

**Affiliations:** 1 School of Information and Communication Engineering, Beijing University of Posts and Telecommunications, Beijing 100876, China; 2 Beijing Laboratory of Advanced Information Network, Beijing University of Posts and Telecommunications, Beijing 100876, China

**Keywords:** intelligent reflecting surface, unmanned aerial vehicle communication, secrecy capacity maximization, convex optimization algorithm

## Abstract

As a new technology for reconstructing communication environments, intelligent reflecting surfaces (IRSs) can be applied to UAV communication systems. However, some challenges exist in IRS-assisted UAV communication system design, such as physical layer security issues, IRS design, and power consumption issues owing to the limitation of the hardware. Therefore, a secrecy capacity optimization scheme for an active IRS-assisted unmanned aerial vehicle (UAV) communication system is proposed to solve multi-user security issues. In particular, controllable power amplifiers are integrated into reflecting units to solve the problem of blocked links, and the UAV can dynamically select the served user according to the channel quality. In order to maximize the system average achievable secrecy capacity and ensure the power constraints of the UAV and active IRS, user scheduling, UAV trajectory, beamforming vector, and reflection matrix are jointly optimized, and the block coordinate descent (BCD) algorithm is applied to solve this non-convex problem. Simulation results show that the active IRS-assisted UAV communication scheme can significantly weaken the “multiplicative fading” effect and enhance the system secrecy capacity by 55.4% and 11.9% compared with the schemes with passive IRS and without optimal trajectory, respectively.

## 1. Introduction

In wireless communication systems, electromagnetic waves experience different kinds of unpredictable changes in propagating environments, which is difficult to explain and is always considered to be probabilistic. Due to obstacles in the propagation environment, especially in urban areas, the signals will not only be affected by free-space path loss but also be reflected, refracted and scattered, and so on, which will eventually result in significant effects on wireless communication performance. Thus, the controlled meta-surfaces technique is invented to programmatically control the behavior of wireless environments [1]. Meanwhile, it has been proved that programmable meta-surfaces can reshape the amplitude and phase of electromagnetic waves efficiently in real time thanks to the concept of intelligent reflecting surfaces (IRSs) [2]. However, traditional passive IRSs can only achieve negligible gains due to the “multiplicative fading” effect [3].

Unmanned aerial vehicles (UAVs) can effectively improve wireless networks’ throughput by making full use of the benefits of line-of-sight (LoS) link transmission and flight flexibility. Moreover, it is anticipated to be a key component of fifth-generation mobile networks to meet the requirement of special scenarios and ubiquitous access [4]. However, in complicated wireless propagation environments, especially in urban areas, the LoS links tend to experience severe deterioration due to the blockage of dense buildings and trees [5]. Fortunately, these challenges can be overcome by deploying IRS in the system since large-scale IRS reflecting elements can jointly beamform the signals in a desired direction by adjusting the IRSs parameters [6,7,8].

In addition, the channel response of the legitimate users and the eavesdropper is strongly correlated with the increasing number of access users, which brings a severe physical layer security problem. Saba et al. [9] investigated the secrecy rate in IRS-assisted multi-user multiple-input multiple-output (MIMO) systems and proposed two low-complexity iterative algorithms, namely two-tier and single-loop forms. Zhang et al. [10] studied the energy effectiveness of an active IRS-aided multiple-input single-output (MISO) secure system, assessed the system’s energy cost quantitatively, and developed a joint optimization strategy to reduce the energy cost while adhering to the secrecy rate limitation.

Integrating IRS technology into UAV-enabled communication systems can provide intelligent compensation for path loss, reduce interference and build energy-efficient, secure, and robust air-to-ground communications [11]. Saxena et al. [12] investigated the effects of jamming caused by a malicious UAV on the performance of a free-space optical communication system, where a legitimate UAV served as a relay and an IRS was designed to improve the quality of received signals and enlarge the coverage. The overall average bit error rate and outage probability with non-Gaussian additive noise were derived and analyzed. Han et al. [13] proposed a UAV-empowered IRS-backscatter communications network, where a passive IRS acts as the backscatter device and uses the received signals for backscatter communications to guarantee secure transmission. Tang et al. [14] presented an anti-eavesdropping communication scheme to exploit aerial active reflecting and jamming to enhance wireless security in the presence of channel uncertainties at the eavesdroppers. Specifically, a robust optimization approach was employed to tackle the reflecting and jamming designing problem, and the aerial deployment was obtained through deep reinforcement learning (DRL). To increase the secrecy capacity, Pang et al. [15] considered secure transmission issues in IRS-assisted UAV communications by joint optimization of UAV trajectory, beamforming vector, reflecting matrix, etc. However, this research work only took the single-user service case into account, but not for multiple users. In [16], a secure IRS-assisted UAV wireless communication system based on multiple users was proposed. The passive beamforming, ground user association, UAV flight trajectory, and transmit power were jointly optimized to maximize the minimum average secrecy rate of ground users. However, the deployment of passive IRS limited the system security performance improvement.

Additionally, investigations in [6,17] illustrated that UAV-mounted IRS can achieve superior performance and flexibility compared to traditional fixed IRS. A downlink multi-user MISO flying IRS system model was considered in [18]. Additionally, a DRL algorithm named FlyReflect was proposed to jointly optimize the flying trajectory and IRS phase-shift matrix. Truong et al. formulated an optimization problem to maximize the achievable system sum rate by jointly optimizing the flight trajectory and phase-shift matrix of the IRS, and a DRL method was applied to solve it. Nguyen et al. [19] proposed an approach of low complexity for extending network coverage in a massive MIMO communication network, where multiple UAV-mounted IRSs were deployed. Moreover, a DRL method is adopted to jointly optimize the power coefficients and the phase shifts of the multiple IRSs. However, attaching IRSs to the UAV will increase the energy consumption of the UAV. Considering a large reflecting surface, the vibration and wind resistance of the UAV cannot be neglected.

To overcome the “multiplicative fading” effect introduced by passive IRSs, the power amplifier can be integrated into its reflecting units to amplify the reflected signals [20]. Zhang et al. [21] analyzed the performance of active IRSs and demonstrated the effectiveness of active IRSs for the first time. However, the current fully connected architecture of the active IRS consumes additional power, since there are abundant integrated power amplifiers. In contrast to the fully connected architecture, Liu et al. [22] presented a sub-connected architecture in which several units share a single power amplifier and individually regulate their phase shift, considerably reducing the number of power amplifiers, and the new architecture can achieve improved energy efficiency. In [23], a low-complexity approximated joint precoding algorithm based on alternating optimization was proposed to optimize the beamforming and active reflector matrix, which further demonstrated the practicability of active intelligent reflection in secure transmission. However, the amplification of noise by its active IRS was neglected, which makes the optimal solution inconsistent with the actual solution. Nguyen et al. [24] considered a novel hybrid active/passive IRS-assisted UAV communications system. The hybrid IRS was equipped with a few active elements, which not only reflect but also amplify the incident signals for significant performance improvement. To maximize the minimum rate among users, the location, and power allocation of the UAV and the IRS reflecting/amplifying coefficients were jointly optimized. The application of hybrid IRS enables improved system performance without additional power consumption. For better comparison, the crucial parameters and optimization methods of reviewed works [13,14,15,16,23,24] are provided in Table 1.

In general, there has been a fair amount of study on IRS-assisted UAV secure communication systems, but rare studies have considered both active IRS adjustment and multi-user services at the same time. The authors of [14,24] only discuss the location of UAVs, without considering the impact of UAV trajectory on the communication environment. In addition, few works have considered both the use of active IRSs and relevant energy constraints. Therefore, an active IRS-assisted UAV multi-user communication system is established, where multiple users are deployed on the ground and time division multiple access (TDMA) is applied as an access scheme to the network. Specifically, in urban scenarios with dense buildings and trees, where the LoS links between the UAV and ground users are often blocked, we deploy active IRS on building surfaces to enhance system security. With the objective of maximizing the average secrecy capacity, which can be represented by the average secrecy rate, user scheduling, UAV trajectory, multi-antenna beamforming, and the amplitude and phase of IRS are jointly optimized. The main contributions of this paper are summarized as follows:

- We present an IRS-assisted UAV multi-user communication system model, where the UAV can dynamically select the best user for service within each time slot according to its channel conditions. Considering the presence of passive eavesdroppers and the LoS link being blocked between the UAV and legitimate users, the user scheduling, UAV trajectory, beamforming vector, and the reflecting matrix of IRS are jointly optimized to maximize the average achievable secrecy capacity during the flight.
- The formulated non-convex optimization problem is first divided into four sub-problems. However, the sub-problems are still challenging to solve due to fractional and non-convex objectives. Hence, successive convex approximation (SCA) technology is applied in this paper to transform the trajectory optimization sub-problem into a convex form. Furthermore, the sub-problems of beam design and IRS control are transformed into a convex problem by applying the Charnes–Cooper transformation (CCT) method and the majorization-minimization (MM) algorithm, respectively. In order to tackle the aforementioned sub-problems and obtain the sub-optimal solution, we finally introduce the block coordinate descent (BCD) approach.
- Simulation results validate the effectiveness of the proposed scheme and show that the secrecy capacity increased compared with the schemes with passive IRS and without optimal trajectory, respectively. It is shown that the active IRS-aided UAV scheme is efficient in reducing the impact of the “multiplicative fading” effect in secure communication systems.

## 2. System Model

**Figure 1:**
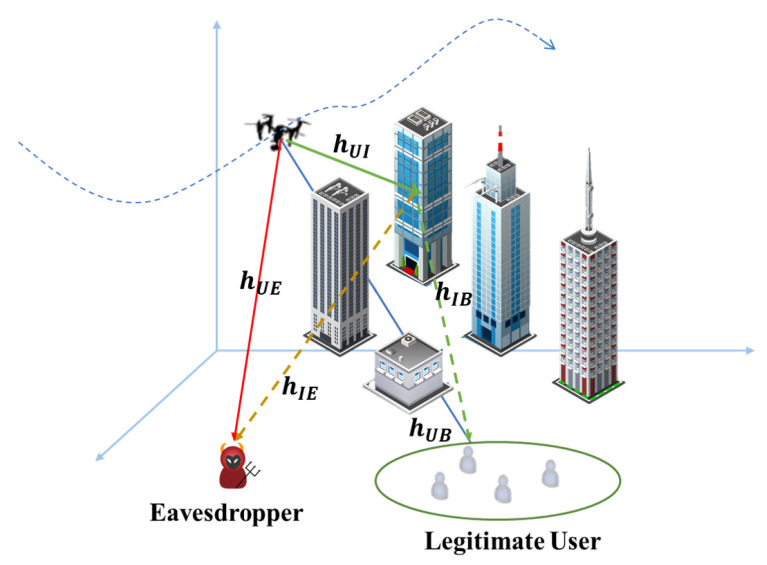
The model of the active IRS-assisted UAV secure communication system.

Figure 1 depicts a model of an active IRS-assisted UAV secure communication system. The UAV serves multiple users as a mobile base station, but a passive eavesdropper exists in the environment at the same time. In addition, we assume that the UAV can access the position of both legitimate users and eavesdroppers based on infrared detection. For clarity, the utilized notations are summarized in Table 2.

### 2.1. Channel Model

A three-dimensional Cartesian coordinate system is established, *J* ground legitimate users and a single passive eavesdropper are distributed in a stationary area. Let the horizontal coordinate of the *j*-th legitimate user Bj and active IRS be $w_{Bj}=\left[x_{Bj},y_{Bj}\right]$ and $w_{I}=\left[x_{I},y_{I}\right]$, respectively. Based on infrared detection, the UAV can obtain the eavesdropper’s location $w_{E}=\left[x_{E},y_{E}\right]$. This paper adopts the access mode of TDMA and the full cycle time T is discretized into N equal time slots, i.e., T=Nδ, where δ is the length of the unit time slot and multiple legitimate users will occupy different time slots. Since the time slot is small enough, the position of the UAV can be considered unchanged [25]. In addition, it is assumed that IRS has a fixed height $H_{I}$ and the UAV’s flight height is fixed at $H_{U}$. The horizontal position of the UAV in the *n*-th time slot can be stated as $q\left[n\right]={\left[x_{u}\left[n\right],\;y_{u}\left[n\right]\right]}^{T},n\in N=\left\{1,\;\ldots ,\;N\right\}$. Then, the following constraints need to be satisfied.

(1) $$\|q\left[n+1\right]-q\left[n\right]\|\leq V_{max}\delta ,\;1\leq n\leq N-1,$$

(2) $$q\left[1\right]=q_{I},$$

(3) $$q\left[N\right]=q_{F},$$

where the constraints in (1) state that the maximum flight rate $V_{\max}$ of the UAV restricts the maximum movement of the UAV in δ. The UAV flies with the predetermined initial position $q_{I}$ and final position $q_{F}$. Moreover, the UAV’s vibration can be disregarded because of its small size.

The active IRS is equipped with a uniform plane array (UPA) of K reflecting elements, a controller intelligently changing the phase shift and amplification of each element, while the UAV is equipped with M antennas and both legitimate users and eavesdropper are equipped with a single omnidirectional antenna. Let the phase-shift matrix $\Theta \left[n\right]=diag\left\{e^{j\theta_{1}\left[n\right]},\ldots ,e^{j\theta_{K}\left[n\right]}\right\}$, where $\theta_{k}\left[n\right]\in \left[0,2\pi \right)\;,\;k\in K=\left\{1,\ldots ,K\right\}$. The enhanced signal by the active IRS can be expressed as:

(4) $$y=\underset{\mathrm{desired}\;\mathrm{signal}\;}{\underset{︸}{P\Theta x}}+\underset{dynamic\;\mathrm{noise}}{\underset{︸}{P\Theta v}}+\underset{static\;\mathrm{noise}}{\underset{︸}{n_{s}}},$$

where x is the transmitted signal and $P=diag\left(p_{1},\cdots ,p_{K}\right)$ denotes the amplification factor matrix. Thanks to the integrated reflection-type amplifier, each element $p_{k}$ can be larger than one but not larger than $\eta_{k}$. It can be seen from (4) that the reflector amplifies not only the useful signal x but also the noise v, it will bring new static noise $n_{s}$. Considering that PΘ always appears in a coupled fashion, let $\psi =P\Theta ={\left[p_{1}e^{j\theta_{1}},\cdots ,p_{K}e^{j\theta_{K}}\right]}^{H}$, Ψ=diag(ψ). Assuming the UAV serves only one user in a time slot, $\alpha_{j}\left[n\right]=1$ indicates that Bj is served in the *n*-th time slot, otherwise $\alpha_{j}\left[n\right]=0$, we can obtain the following constraints:

(5) $$\alpha_{j}\;\left[n\right]\in \left\{0,1\right\},\forall n,j,$$

(6) $$\overset{J}{\underset{j=1}{\sum }}\alpha_{j}\left[n\right]\leq 1,\forall n,1\leq j\leq J.$$

We assume that the channel quality predominantly depends on the UAV–IRS distance since the air-to-ground communication channels are mainly dominated by the LoS links [26]. Particularly, it is presumed that the receivers will properly compensate for the Doppler effect brought on by the UAV’s motion [27]. Unlike a uniform linear array (ULA) at the UAV, we utilize a UPA at the IRS in this paper. Additionally, the active IRS consists of $N=N_{y}\times N_{z}$ elements and UAV is equipped with M antennas. The total array response of the corresponding channel can be seen as the product of the array response of receivers and that of the transmitter [28]. Therefore, the channel modeling is characterized as the product channels [7].

(7) $$H_{UI}\left[n\right]=\underset{path\;loss}{\underset{︸}{\sqrt{\rho_{0}d_{UI}^{-2}\left[n\right]}}}\underset{array\;response}{\underset{︸}{a_{N}^{H}\left(\theta_{\mathrm{AoA}}\left[n\right],\eta_{\mathrm{AoA}}\left[n\right]\right)a_{M}\left(\gamma_{\mathrm{AoD}}\left[n\right]\right)}}$$

(8) $$a_{N}\left(\theta ,\eta \right)=\;\left[1,\ldots ,e^{j\frac{2\pi d_{0}}{\lambda }\left(\left(n_{1}-1\right)\cos(\eta )\sin(\theta )+\left(n_{2}-1\right)\sin(\eta )\right)},\ldots ,e^{j\frac{2\pi d_{0}}{\lambda }\left(\left(N_{y}-1\right)\cos(\eta )\sin(\theta )+\left(N_{z}-1\right)\sin(\eta )\right)}\right]$$

(9) $$a_{M}\left(\gamma \right)=\left[1,e^{j2\pi \frac{d}{\lambda }\sin\gamma },\ldots ,e^{j2\pi \frac{d}{\lambda }\left(M-1\right)\sin\gamma }\right],$$

where $\rho_{0}$ denotes the channel gain at the reference distance $D_{0}=1m$, assuming the path loss exponent related to the U−*I* link is 2, $d_{UI}\left[n\right]=\sqrt{\|q\left[n\right]-w_{I}\|{}^{2}+{\left(H_{U}-H_{I}\right)}^{2}}$ denotes the distance between the UAV and the active IRS in the *n*-th time slot, $a_{N}\left(\theta ,\eta \right)$ and $a_{M}\left(\gamma \right)$ are the array responses of IRS and transmitter of UAV, λ is the wavelength, d is the antenna separation, and $d_{0}$ is the separation of elements at the IRS. $\theta_{\mathrm{AoA}}\left[n\right]$ and $\eta_{\mathrm{AoA}}\left[n\right]$ represent the azimuth and elevation angle of arrival (AoA) of the signal from the UAV to the IRS in the *n*-th time slot, respectively. Additionally, $\gamma_{\mathrm{AoA}}\left[n\right]$ denotes the angle of departure (AoD) associated with the UAV in the *n*-th time slot.

In contrast to $H_{UI}\left[n\right]$, the channel from IRS to the legitimate users and eavesdropper contains both the LoS component and the non-LoS (NLoS) component. Therefore, the Rician fading channel model [3,7,13] is adopted, the channel gain $h_{IBj}\left[n\right]$ can be expressed as

(10) $$h_{IBj}\left[n\right]=\sqrt{\rho_{0}d_{IBj}^{-\alpha }\left[n\right]}\left(\sqrt{\frac{\kappa }{1+\kappa }}h_{IBj}^{\mathrm{LoS}}+\sqrt{\frac{1}{\kappa +1}}h_{IBj}^{\mathrm{NLoS}}\left[n\right]\right),$$

where α is the path loss exponent corresponding to the I−Bj link, $d_{IBj}=\sqrt{\|w_{I}-w_{Bj}\|{}^{2}+H_{I}^{2}}$ denotes the distance between the active IRS and Bj, and κ is the Rician factor. The LoS component can be calculated as $h_{IBj}^{\mathrm{LoS}}=a_{N}^{H}\left(\theta_{\mathrm{AoD}},\eta_{\mathrm{AoD}}\right)$ and the NLoS component $h_{IBj}^{\mathrm{NLoS}}\left[n\right]$ is the random scattering component independently modeled by a zero mean and a unit-variance circularly symmetric complex Gaussian (CSCG) random variable. $h_{IE}\left[n\right]$ can be modeled in the same way.

In actual implementations, copious obstructions in the complicated urban environment may hinder the LoS path of the UAV to the user, while the wireless channel is still replete with extensive scatters [29]. The channel coefficients of U−Bj/E link are given by

(11) $$h_{UBj}\left[n\right]=\sqrt{\rho_{0}d_{UBj}^{-\beta }\left[n\right]}\tilde{h}\left[n\right],$$

(12) $$h_{UE}\left[n\right]=\sqrt{\rho_{0}d_{UE}^{-\beta }\left[n\right]}\tilde{h}\left[n\right],$$

where β is the path loss exponent of U−Bj/EVE link, $d_{UBj}\left[n\right]=\sqrt{\|q\left[n\right]-w_{Bj}\|{}^{2}+H_{U}^{2}}$ and $d_{UE}\left[n\right]=\sqrt{\|q\left[n\right]-w_{E}\|{}^{2}+H_{U}^{2}}$ denotes the distance from the UAV to the served user and passive eavesdropper, respectively. $\tilde{h}\left[n\right]$ is modeled as a CSCG distribution with zero mean and unit variance.

### 2.2. Secrecy Capacity Model

The received signal of the legitimate user Bj and the eavesdropper in the *n*-th time slot can be given as follows, respectively.

(13) $$y_{Bj}\left[n\right]=\left(h_{UBj}\left[n\right]+h_{IBj}^{H}\left[n\right]\Psi \left[n\right]H_{UI}\left[n\right]\right)w\left[n\right]x\left[n\right]+h_{IB}\Psi n_{I}+n_{Bj},$$

(14) $$y_{E}\left[n\right]=\left(h_{UE}\left[n\right]+h_{IE}^{H}\left[n\right]\Psi \left[n\right]H_{UI}\left[n\right]\right)w\left[n\right]x\left[n\right]+h_{IE}\Psi n_{I}+n_{E},$$

where x[n] is the transmitted signal; $w\left[n\right]\in {\mathbb{C}}^{M\times 1}$ denotes the beamforming vector at the UAV; $n_{I}\sim CN\left(0,\sigma_{I}^{2}\right)$, $n_{B}\sim CN\left(0,\sigma_{B}^{2}\right)$, and $n_{E}\sim CN\left(0,\sigma_{E}^{2}\right)$, respectively, represent the noise introduced by IRS, user Bj, and eavesdropper; $\sigma_{I}^{2}$, $\sigma_{B}^{2}$, and $\sigma_{E}^{2}$ represent the noise power. The SINR of the legitimate user Bj and the eavesdropper in the *n*-th time slot, respectively, are given as:

(15) $$\gamma_{Bj}\left[n\right]=\frac{{\left|\left(h_{UBj}\left[n\right]+h_{IBj}^{H}\left[n\right]\Psi \left[n\right]H_{UI}\left[n\right]\right)w\left[n\right]\right|}^{2}}{\sigma_{B}^{2}+\|h_{IBj}^{H}\left[n\right]\Psi {\left[n\right]\|}^{2}\sigma_{I}^{2}},$$

(16) $$\gamma_{E}\left[n\right]=\frac{{\left|\left(h_{UEj}\left[n\right]+h_{IE}^{H}\left[n\right]\Psi \left[n\right]H_{UI}\left[n\right]\right)w\left[n\right]\right|}^{2}}{\sigma_{E}^{2}+\|h_{IE}^{H}\left[n\right]\Psi {\left[n\right]\|}^{2}\sigma_{I}^{2}},$$

The static noise $n_{s}$ introduced by the IRS in (4) is independent of Ψ and relatively small, and can be omitted. Hereto, the system’s average secrecy capacity during flight time can be described as

(17) $$R_{\sec}=\frac{1}{N}\overset{N}{\underset{n=1}{\sum }}{\left[\left(\overset{J}{\underset{j=1}{\sum }}\alpha_{j}\left[n\right]R_{Bj}\left[n\right]\right)-R_{E}\left[n\right]\right]}^{+},$$

where $R_{Bj}\left[n\right]=\log_{2}\left(1+\gamma_{Bj}\left[n\right]\right)$, $R_{E}\left[n\right]=\log_{2}\left(1+\gamma_{E}\left[n\right]\right)$, ${\left[x\right]}^{+}=\max\left(x,0\right)$.

### 2.3. Power Consumption Model

Assuming the UAV flies at a constant power $P_{fly}$, and the total power consumption of UAV in the *n*-th time slot is expressed as follows:

(18) $$P_{UAV}\left[n\right]=\xi {\|w\left[n\right]\|}^{2}+P_{fly},$$

where ξ is the reciprocal of the energy conversion coefficient at the transmitter of the UAV. The maximum transmitting power of the UAV at any time slot during the flight cycle shall not exceed the upper limit of the transmitting power of the antenna $P_{U}$.

(19) $$P_{UAV}\left[n\right]\leq P_{U},\forall n,$$

The active IRS amplifies not only useful signals but also useless ones, thus the total energy consumption of the active IRS in the *n*-th time slot is expressed as

(20) $$P_{IRS}\left[n\right]=\zeta \left(\|\Psi \left[n\right]H_{UI}\left[n\right]w{\left[n\right]\|}^{2}+\|\Psi \left[n\right]{\|}^{2}\sigma_{I}^{2}\right)+NP_{irs}+LP_{amp},$$

where ζ is the reciprocal of the energy conversion coefficient at the transmitter of the active IRS. $P_{irs}$ and $P_{amp}$ constitute the hardware static power of the active IRS, which corresponds to the phase shift and amplifier, respectively. The fully connected architecture is adopted in the active IRS, i.e., L=K. Specifically, $P_{IRS}\left[n\right]$ must not exceed the upper limit of the active IRS $P_{A}$, i.e.,

(21) $$P_{IRS}\left[n\right]\leq P_{A},\forall n.$$

### 2.4. Problem Description

We aim to maximize the average secrecy capacity for legitimate users by jointly designing the user scheduling $S=\left\{\alpha_{j}\left[n\right],n=1,\ldots ,N,j=1,2\ldots J\right\}$, the UAV trajectory Q={q[n],n=1,…,N}, the reflecting matrix of the active IRS Ψ={Ψ[n],n=1,…,N}, and the active beamforming vector W={w[n],n=1,…N}. We also consider the mobility constraints, the power constraints of the active IRS and UAV, and the maximum magnification limit of amplifiers.

The optimization problem (*P*1) is formulated as

(22a) $$\left(P1\right):\underset{S,Q,W,\Psi }{max}R_{sec}=\frac{1}{N}\overset{N}{\underset{n=1}{\sum }}{\left[\left(\overset{J}{\underset{j=1}{\sum }}\alpha_{j}\left[n\right]R_{Bj}\left[n\right]\right)-R_{E}\left[n\right]\right]}^{+}$$

(22b) $$\alpha_{j}\left[n\right]\in \left\{0,1\right\},\forall n,j,$$

(22c) $$\overset{J}{\underset{j=1}{\sum }}\alpha_{j}\left[n\right]\leq 1,\forall n,1\leq j\leq J,$$

(22d) $$P_{UAV}\left[n\right]\leq P_{U},\forall n,$$

(22e) $$P_{IRS}\left[n\right]\leq P_{A},\forall n,$$

(22f) $$\left|\Psi \left[k,k\right]\right|\leq \eta_{k},\forall n,\forall k,$$

(22g) $$q\left[1\right]=q_{I},$$

(22h) $$q\left[N\right]=q_{F},$$

(22i) $$\|q\left[n+1\right]-q\left[n\right]\|\;\leq V_{max}\delta ,\;1\leq n\leq N-1,$$

where $\eta_{k}$ denotes the maximum magnification at the *k*-th reflecting element. The optimization problem (P1) is a non-convex problem and there is a tight coupling among the optimization variables W, Ψ, and Q. In addition, the power constraint and maximum amplification of the IRS should be considered while optimizing the phase factor and amplification factor of the IRS. The channel of the auxiliary communication system of the active IRS is the sum of a direct link and cascade channel, and the active IRS will introduce extra noise. Moreover, the objective function is the difference between the two rates and makes the objective function difficult to solve.

## 3. Joint Optimization Algorithm

**Figure 2:**
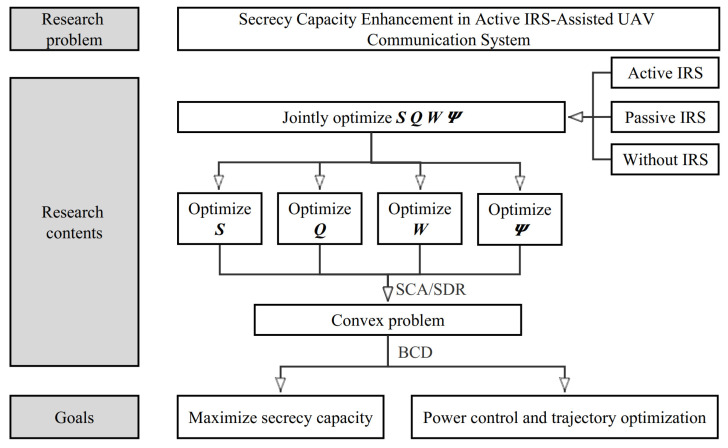
The flow chart of the joint optimization algorithm.

Note that if the value of $R_{\sec}$ is negative in the *n*-th time slot, we can control the transmit beamforming vector w[n]=0, resulting in $R_{\sec}=0$. Thus, by modifying the beamforming vector, we can ensure that the secrecy capacity is never negative. In this case, it is possible to omit the operator ${\left[\;\cdot \;\right]}^{+}$ without affecting the result. Even so, the objective function of the original problem is non-convex and contains non-convex constraints, making it challenging to solve. Considering the coupling relationship among variables, the BCD algorithm is applied to decompose the original problem into four sub-problems: user scheduling, trajectory optimization, beamforming design, and active IRS control. For the first sub-problem, we choose the best user by comparing the overall channel conditions. The locally optimal trajectory solution can be obtained in the second sub-problem. In the beamforming design problem, the variables about beamforming always exist in quadratic terms. We first converted the problem by the semidefinite relaxation (SDR) algorithm and solved the fractional programming problem after transformation by CCT. Similarly, in the IRS designing problem, the SDR algorithm is applied first and the MM algorithm is used to relax the transformed non-convex objective function. The flow chart of the joint optimization algorithm is shown in Figure 2.

### 3.1. User Scheduling Optimization

For the given UAV trajectory Q, beamforming vector W, and reflecting matrix Ψ, the transmission rate of eavesdroppers is settled. Thus, the average secrecy capacity is dependent entirely on the downlink rate of legitimate users. The problem (*P*1) can be rewritten by

(23a) $$\left(P2\right):\underset{S}{max}R_{sec}=\frac{1}{N}\overset{N}{\underset{n=1}{\sum }}\overset{J}{\underset{j=1}{\sum }}\alpha_{j}\left[n\right]R_{Bj}\left[n\right]$$

(23b) (22b), (22c).

By finding $\max(R_{Bc}\left[n\right])$ and setting the corresponding scheduling $\alpha_{c}\left[n\right]=1$, the rest $\alpha_{j}\left[n\right]=0,j\notin c$, then the optimal scheduling can be expressed as

(24) $$\{\begin{array}{ccc} \alpha_{c}\left[n\right]=1 & \alpha_{c}\left[n\right]R_{Bc}\left[n\right]=\max\left(\alpha_{j}\left[n\right]R_{Bj}\right) & \forall j\in J\\ \alpha_{j}\left[n\right]=0 & j\notin c & \end{array}.$$

### 3.2. Trajectory Optimization

With given user scheduling S, beamforming vector W, and reflecting matrix Ψ, the trajectory optimization sub-problem can be formulated as

(25a) $$\left(P3\right):\underset{Q}{max}R_{sec}=\frac{1}{N}\overset{N}{\underset{n=1}{\sum }}\left\{\left(\overset{J}{\underset{j=1}{\sum }}\alpha_{j}\left[n\right]R_{Bj}\left[n\right]\right)-R_{E}\left[n\right]\right\}$$

(25b) (1),(2), (3), (21)

Based on Jensen’s inequality, $R_{E}\left[n\right]$ can be formulated by

$$R_{E}\left[n\right]=E\left[log_{2}\left(1+\varepsilon_{E}\left[n\right]{\left|\left(h_{UE}\left[n\right]+h_{IE}^{H}\left[n\right]\Psi \left[n\right]H_{UI}\left[n\right]\right)\right|}^{2}\right)\right]\leq$$

$$log_{2}\left(1+\varepsilon_{E}\left[n\right]E\left[{\left|\left(h_{UE}\left[n\right]+h_{IE}^{H}\left[n\right]\Psi \left[n\right]H_{UI}\left[n\right]\right)\right|}^{2}\right]\right)=$$

(26) $$log_{2}\left(1+\varepsilon_{E}\left[n\right]\left(h_{UE}{\left[n\right]}^{2}+{\left|h_{IE}^{H}\left[n\right]\Psi \left[n\right]H_{UI}\left[n\right]\right|}^{2}\right)\right)={\hat{R}}_{E}\left[n\right].$$

Considering that W, S, and Ψ are fixed, let $A_{1}=\sqrt{\rho_{0}}\left|{\tilde{h}}_{\left[n\right]}\right|,\;A_{2}\left[n\right]=\sqrt{\rho_{0}}\left|h_{IBj}\left[n\right]\Psi \left[n\right]{\tilde{H}}_{UI}\left[n\right]\right|,$ $A_{3}\left[n\right]=\sqrt{\rho_{0}}\left|h_{IE}\left[n\right]\Psi \left[n\right]{\tilde{H}}_{UI}\left[n\right]\right|$, ${\tilde{H}}_{UI}\left[n\right]=a_{N}^{H}\left(\theta_{\mathrm{AoA}}\left[n\right],\eta_{\mathrm{AoA}}\left[n\right]\right)a_{M}\left(\gamma_{\mathrm{AoD}}\left[n\right]\right)$, $\varepsilon_{Bj}\left[n\right]=\frac{{\left|w\left[n\right]\right|}^{2}}{\|h_{IBj}^{H}\left[n\right]\Psi {\left[n\right]\|}^{2}\sigma_{I}^{2}+\sigma_{B}^{2}}$, $\varepsilon_{E}\left[n\right]=\frac{w{\left[n\right]}^{2}}{\|h_{IBj}^{H}\left[n\right]\Psi {\left[n\right]\|}^{2}\sigma_{I}^{2}+\sigma_{E}^{2}},\;\forall n$.

Then, the simplified transmission rate of them can be expressed as

(27) $$R_{Bj}\left[n\right]=log_{2}\left(1+\varepsilon_{Bj}\left[n\right]\left(\frac{A_{1}^{2}}{d_{UBj}^{\beta }\left[n\right]}+\frac{A_{2}^{2}\left[n\right]}{d_{UI}^{2}\left[n\right]}+\frac{2A_{1}A_{2}\left[n\right]}{d_{UBj}^{\frac{\beta }{2}}d_{UI}\left[n\right]}\right)\right),$$

(28) $${\hat{R}}_{E}\left[n\right]=log_{2}\left(1+\varepsilon_{E}\left[n\right]\left(\frac{A_{1}^{2}}{d_{UE}^{\beta }\left[n\right]}+\frac{A_{3}^{2}\left[n\right]}{d_{UI}^{2}\left[n\right]}\right)\right).$$

However, it is difficult to deal with (27) and (28) optimally due to their non-convexity. We introduce slack variables $\nu \left[n\right]=d_{UBj}\left[n\right]$, ν={ν[n],∀n}, and $\omega \left[n\right]=d_{UI}\left[n\right]$, ω={ω[n],∀n}. Provided that ${\hat{R}}_{E}\left[n\right]$ is always negative, which is hard to solve, we introduce slack variables ζ[n], $\;\mu_{t}\left[n\right]\left(t=1,\;2\right)$, the constraints can be expressed as

(29) $$\zeta \left[n\right]\geq log_{2}\left(1+A_{1}^{2}\varepsilon_{E}\left[n\right]e^{\mu_{1}\left[n\right]}+A_{3}^{2}\left[n\right]\varepsilon_{E}\left[n\right]e^{\mu_{2}\left[n\right]}\right),\;\zeta =\left\{\zeta \left[n\right],\forall n\right\},$$

(30) $$e^{-\mu_{1}\left[n\right]}\leq \|q\left[n\right]-w_{E}\|{}^{2}+H_{U}^{2},\;\mu_{1}=\left\{\mu_{1}\left[n\right],\forall n\right\},$$

(31) $$e^{-\mu_{2}\left[n\right]}\leq \|q\left[n\right]-w_{I}\|{}^{2}+{\left(H_{U}-H_{I}\right)}^{2},\;\mu_{2}=\left\{\mu_{2}\left[n\right],\forall n\right\}.$$

Then, $R_{Bj}\left[n\right]$ can be reformulated as follows:

(32) $$R_{Bj}\left[n\right]=log_{2}\left(1+\varepsilon_{Bj}\left[n\right]\left(\frac{A_{1}^{2}}{\nu^{\beta }\left[n\right]}+\frac{A_{2}^{2}\left[n\right]}{\omega^{2}\left[n\right]}+\frac{2A_{1}A_{2}\left[n\right]}{\nu^{\frac{\beta }{2}}\left[n\right]\omega \left[n\right]}\right)\right).$$

The original sub-problem can be written as

(33a) $$\left(P3.1\right):\underset{Q,\mu_{1},\mu_{2},\nu ,\omega ,\zeta }{max}\frac{1}{N}\overset{N}{\underset{n=1}{\sum }}\left(\overset{J}{\underset{j=1}{\sum }}\alpha_{j}\left[n\right]R_{Bj}\left[n\right]-\zeta \left[n\right]\right)$$

(33b) $$d_{UBj}\left[n\right]\leq \nu \left[n\right],\forall n,$$

(33c) $$d_{UI}\left[n\right]\leq \omega \left[n\right],\forall n,$$

(33d) (29), (30),(31),

which is still non-convex as $R_{Bj}\left[n\right]$ is still concave with respect to $\left\{\nu_{k}\left[n\right],\forall n\right\}$ and $\left\{\omega_{k}\left[n\right],\forall n\right\}$. The SCA technique is applied to relax the aforementioned issues to its global lower-bound. The first-order Taylor expansion of (32) can be used to approximate it at the supplied local points $\left\{\nu_{k}\left[n\right],\forall n\right\}$ and $\left\{\omega_{k}\left[n\right],\forall n\right\}$ can be given by

(34) $$R_{Bj}^{*}\left[n\right]\geq R_{Bj}^{lb}\left[n\right]=log_{2}G_{1}\left[n\right]+\frac{G_{2}\left[n\right]}{G_{1}\left[n\right]\ln2}\left(\nu \left[n\right]-\nu_{k}\left[n\right]\right)\;\;+\frac{G_{3}\left[n\right]}{G_{1}\left[n\right]\ln2}\left(\omega \left[n\right]-\omega_{k}\left[n\right]\right),$$

(35) $$G_{1}\left[n\right]=1+\varepsilon_{Bj}\left[n\right]\left(\frac{A_{1}^{2}}{\nu_{k}^{\beta }\left[n\right]}+\frac{A_{2}^{2}\left[n\right]}{\omega_{k}{}^{2}\left[n\right]}+\frac{2A_{1}A_{2}\left[n\right]}{\nu_{k}^{\frac{\beta }{2}}\left[n\right]\omega_{k}\left[n\right]}\right),$$

(36) $$G_{2}\left[n\right]=-\varepsilon_{Bj}\left[n\right]\left(\frac{A_{1}^{2}}{\nu_{k}^{\beta +1}\left[n\right]}+\frac{A_{1}A_{2}\left[n\right]}{\nu_{k}^{\frac{\beta }{2}+1}\left[n\right]\omega_{k}\left[n\right]}\right),$$

(37) $$G_{3}\left[n\right]=-\varepsilon_{Bj}\left[n\right](\frac{A_{2}^{2}\left[n\right]}{\omega_{k}^{3}\left[n\right]}+\frac{A_{1}A_{2}\left[n\right]}{\nu_{k}{}^{\frac{\beta }{2}}\left[n\right]\omega_{k}^{2}\left[n\right]}).$$

Moreover, the constraints (33b) and (33c) are convex with respect to $\left\{\nu_{k}\left[n\right],\forall n\right\}$ and $\left\{\omega_{k}\left[n\right],\forall n\right\}$, and the right parts in (30) and (31) are convex with respect to q[n], and can also be relaxed to its lower-bound by the first-order Taylor expansion, which can be given by

(38) $$v^{2}\left[n\right]\geq 2\nu_{k}\left[n\right]\nu \left[n\right]-\nu_{k}^{2}\left[n\right],\forall n,$$

(39) $$\omega^{2}\left[n\right]\geq 2\omega_{k}\left[n\right]\omega \left[n\right]-\omega_{k}^{2}\left[n\right],\forall n$$

(40) $$u_{1}^{*}\left[n\right]\geq \|q^{k}\left[n\right]-w_{E}\|{}^{2}+H_{U}^{2}+2{\left(q^{k}\left[n\right]-w_{E}\right)}^{T}\left(q\left[n\right]-q^{k}\left[n\right]\right),\forall n,$$

(41) $$u_{2}^{*}\left[n\right]\geq \|q^{k}\left[n\right]-w_{I}\|{}^{2}+H_{U}^{2}+2{\left(q^{k}\left[n\right]-w_{I}\right)}^{T}\left(q\left[n\right]-q^{k}\left[n\right]\right),\forall n.$$

Bringing them to (33b), (33c), (30), and (31), respectively, the problem (P3.1) can be approximated as

(42a) $$\left(P3.2\right):\underset{Q,\mu_{1},\mu_{2},\nu ,\omega ,\zeta }{max}\frac{1}{N}\overset{N}{\underset{n=1}{\sum }}\left(\overset{J}{\underset{j=1}{\sum }}\alpha_{j}\left[n\right]R_{Bj}^{*}\left[n\right]-\zeta \left[n\right]\right)$$

(42b) $$d_{UBj}^{2}\left[n\right]+\nu_{k}^{2}\left[n\right]-2\nu_{k}\left[n\right]\nu \left[n\right]\leq 0,\;\forall n,$$

(42c) $$d_{UI}^{2}\left[n\right]+\omega_{k}^{2}\left[n\right]-2\omega_{k}\left[n\right]\omega \left[n\right]\leq 0,\;\forall n,$$

(42d) $$e^{-\mu_{1}\left[n\right]}\leq u_{1}^{*}\left[n\right],\;\forall n,$$

(42e) $$e^{-\mu_{2}\left[n\right]}\leq u_{2}^{*}\left[n\right],\;\forall n,$$

(42f) (1),(2), (3), (21),(29).

Thus far, (P3.2) is a standard convex problem that can be solved with the CVX tool.

### 3.3. Beamforming Optimization

Given user scheduling S, UAV trajectory Q, and reflecting matrix Ψ, the beamforming design sub-problem can be expressed as

(43a) $$\left(P4\right):\underset{W}{max}R_{sec}=\frac{1}{N}\overset{N}{\underset{n=1}{\sum }}\left(\overset{J}{\underset{j=1}{\sum }}\alpha_{j}\left[n\right]R_{Bj}\left[n\right]-R_{E}\left[n\right]\right)$$

(43b) (19),(21).

Since the cascaded channel of the problem is extremely complex and the fixed power consumption in the constraint is known, the fixed parts can be given by (44)–(49):

(44) $${\tilde{P}}_{U}\left[n\right]=\frac{1}{\xi \left(P_{U}\left[n\right]-P_{fly}\right)},\forall n,$$

(45) $${\tilde{P}}_{A}\left[n\right]=\frac{1}{\zeta \left(P_{A}\left[n\right]-NP_{irs}-LP_{amp}\right)}-{\left\|\Psi \left[n\right]\right\|}^{2}\sigma_{I}^{2},\forall n,$$

(46) $${\tilde{h}}_{B}\left[n\right]=\frac{h_{B}\left[n\right]}{\sqrt{\sigma_{B}^{2}+{\|h}_{IBj}^{H}\left[n\right]\Psi {\left[n\right]\|}^{2}\sigma_{I}^{2}}},\forall n,$$

(47) $${\tilde{h}}_{E}\left[n\right]=\frac{h_{E}\left[n\right]}{\sqrt{\sigma_{E}^{2}+{\|h}_{IE}^{H}\left[n\right]\Psi {\left[n\right]\|}^{2}\sigma_{I}^{2}}},\forall n,$$

(48) $$h_{B}\left[n\right]=h_{UBj}\left[n\right]+h_{IBj}^{H}\left[n\right]\Psi \left[n\right]H_{UI}\left[n\right],\forall n,$$

(49) $$h_{E}\left[n\right]=h_{UE}\left[n\right]+h_{IE}^{H}\left[n\right]\Psi \left[n\right]H_{UI}\left[n\right],\forall n.$$

Since the log formula is monotonous, the solution of the original problem will not be changed by omitting it, then the original sub-problem can be expressed as

(50a) $$\left(P4.1\right):\underset{W}{max}\frac{1}{N}\overset{N}{\underset{n=1}{\sum }}\left(\left(1+{\left|{\tilde{h}}_{B}\left[n\right]w\left[n\right]\right|}^{2}\right){\left(1+{\left|{\tilde{h}}_{E\left[n\right]}w\left[n\right]\right|}^{2}\right)}^{-1}\right)$$

(50b) $${\|w\left[n]\right\|}^{2}\leq {\tilde{P}}_{U}\left[n\right],\forall n,$$

(50c) $$\|\Psi \left[n\right]H_{UR}\left[n\right]w{\left[n\right]\|}^{2}\leq {\tilde{P}}_{A}\left[n\right],\forall n.$$

(P4.1) is testing due to the non-convex objective function. Inspired by the SDR technique, let $Y\left[n\right]=w\left[n\right]w^{H}\left[n\right]$, Y={Y[n],∀n}, the original sub-problem can be transferred to a relaxed one:

(51a) $$\left(P4.2\right):\underset{Y}{max}\frac{1}{N}\overset{N}{\underset{n=1}{\sum }}\left(\left(1+{\tilde{h}}_{B}\left[n\right]Y\left[n\right]{\tilde{h}}_{B}^{H}\left[n\right]\right){\left(1+{\tilde{h}}_{E}\left[n\right]Y\left[n\right]{\tilde{h}}_{E}^{H}\left[n\right]\right)}^{-1}\right)$$

(51b) Y[n]≽0,∀n,

(51c) $$tr(Y\left[n\right])\leq {\tilde{P}}_{U}\left[n\right],\forall n,$$

(51d) $$tr\left(\Psi \left[n\right]H_{UI\left[n\right]}Y\left[n\right]H_{UI}^{H}\left[n\right]\Psi {\left[n\right]}^{H}\right)\leq {\tilde{P}}_{A}\left[n\right],\forall n,$$

which is a standard fractional programming problem. By applying the CCT method [30], the above problem can be converted to

(52a) $$\left(P4.3\right):\underset{\tilde{Y},t}{max}\;\;\frac{1}{N}\overset{N}{\underset{n=1}{\sum }}\left(t\left[n\right]+{\tilde{h}}_{B}\left[n\right]\tilde{Y}\left[n\right]{\tilde{h}}_{B}^{H}\left[n\right]\right)$$

(52b) $$tr(\tilde{Y}\left[n\right])\leq t\left[n\right]{\tilde{P}}_{U}\left[n\right],\forall n,$$

(52c) $$tr\left(\Psi \left[n\right]H_{UI}\left[n\right]\tilde{Y}\left[n\right]H_{UI}^{H}\left[n\right]\Psi {\left[n\right]}^{H}\right)\leq t\left[n\right]{\tilde{P}}_{A}\left[n\right],\forall n,$$

(52d) $${\tilde{h}}_{E}\left[n\right]\tilde{Y}\left[n\right]{\tilde{h}}_{E}^{H}\left[n\right]+t\left[n\right]=1,\forall n,$$

(52e) t[n]≥0,∀n,

(52f) $$\tilde{Y}\left[n\right]≽0,\forall n,$$

where $\tilde{Y}\left[n\right]=t\left[n\right]Y\left[n\right]$, $\tilde{Y}=\left\{\tilde{Y}\left[n\right],\forall n\right\}$, $t\left[n\right]=1/\left(1+{\tilde{h}}_{E}\left[n\right]Y{\tilde{h}}_{E}^{H}\left[n\right]\right)$, and t={t[n],∀n}, (P4.3) can be properly optimized via the CVX tool. However, rank(Y[n])=1 may not be satisfied, so the original w[n] cannot be recovered. Specifically, the rank-1 constraint can be equivalently expressed as $tr\left(\tilde{Y}\left[n\right]\right)-\lambda_{max}\left(\tilde{Y}\left[n\right]\right)\leq 0$, where $\lambda_{\max}\left(X\right)$ is the largest eigenvalue of X, tr(X) represents the eigenvector corresponding to the largest eigenvalue of X. By constructing the penalty function, the objective function of this sub-problem is converted as follows:

(53) $$\frac{1}{N}\overset{N}{\underset{n=1}{\sum }}\left(-\left(t\left[n\right]+{\tilde{h}}_{B}\left[n\right]\tilde{Y}\left[n\right]{\tilde{h}}_{B}^{H}\left[n\right]\right)+\Lambda_{w}\left[n\right]\left(tr(\tilde{Y}\left[n\right])-\lambda_{max}\left(\tilde{Y}\left[n\right]\right)\right)\right).$$

When $\Lambda_{w}\left[n\right]$ is large enough, we have $tr\left(\tilde{Y}\left[n\right]\right)-\lambda_{max}\left(\tilde{Y}\left[n\right]\right)\approx 0$, then the rank-1 constraint can be satisfied. However, (53) is still concave, and $\lambda_{max}\left(\tilde{Y}\left[n\right]\right)$ is not differentiable. We can apply the sub-gradient of $\lambda_{max}\left(\tilde{Y}\left[n\right]\right)$ as $u_{max}{\left(\tilde{Y}{\left[n\right]}^{\left(r\right)}\right)}^{H}u_{max}\left(\tilde{Y}{\left[n\right]}^{\left(r\right)}\right)$, where $u_{max}\left(X\right)$ is the eigenvector corresponding to the largest eigenvalue of X. Therefore, given a feasible solution $\tilde{Y}{\left[n\right]}^{\left(r\right)}$ for (P4.3) in the *r*-th iteration, we get the improved expression as

(54a) $$\left(P4.4\right):\underset{\tilde{Y},t}{min}\;\frac{1}{N}\overset{N}{\underset{n=1}{\sum }}\left(-\left(t\left[n\right]+{\tilde{h}}_{B}\left[n\right]\tilde{Y}\left[n\right]{\tilde{h}}_{B}^{H}\left[n\right]\right)\right)\;+\frac{1}{N}\overset{N}{\underset{n=1}{\sum }}\left(\Lambda_{w}\left[n\right]\left(tr(\tilde{Y}\left[n\right])-u_{max}{\left(\tilde{Y}{\left[n\right]}^{\left(r\right)}\right)}^{H}\tilde{Y}\left[n\right]u_{max}\left(\tilde{Y}{\left[n\right]}^{\left(r\right)}\right)\right)\right)$$

(54b) (52b), (52c),(52d),(52e),(52f).

Problem (P4.4) can be properly optimized through the CVX solver. Given S, Q, and Ψ, the log function in (P4) can be omitted because of its monotonicity. In objective functions and constraints, the variable W always appears in quadratic form. Adopting the SDR algorithm, the problem is transformed by constructing $Y\left[n\right]=w\left[n\right]w^{H}\left[n\right]$ and the CCT method is used to solve the fractional programming problem (P4.2). It is worth noting that the rank-one constraint is introduced into SDR, so the penalty $\Lambda_{w}$ is constructed to recover the original variable as much as possible. The logic of the penalty-based recovery rank-1 algorithm is shown in Algorithm 1. By iteratively solving problem (P4.4) optimally, we can monotonically tighten the upper bound of (53). Penalty $\Lambda_{w}$ is updated to guarantee the rank-one constraint.

**Algorithm 1** Beamforming Design Algorithm for Problem (P4).(1): Initialization: maximum threshold $\epsilon_{1},\;\epsilon_{2}>0$ penalty $\Lambda_{w}=10$ feasible point $\tilde{Y}{\left[n\right]}^{\left(0\right)}$, $t^{\left(0\right)}$, and number of iterations r = 0.(2): Calculate $\left|tr\left(\tilde{Y}{\left[n\right]}^{\left(r\right)}\right)-\lambda_{max}\left(\tilde{Y}{\left[n\right]}^{\left(r\right)}\right)\right|$. If $|tr\left(\tilde{Y}{\left[n\right]}^{\left(r\right)}\right)-\lambda_{max}\left(\tilde{Y}{\left[n\right]}^{\left(r\right)}\right)|<\epsilon_{1}$, then the algorithm converges, go to step (5), else go to step (3).(3): Optimize $\tilde{Y}{\left[n\right]}^{\left(r+1\right)}$ and $t^{\left(r+1\right)}$ in (P4.4), with given $u_{max}\left(\tilde{Y}{\left[n\right]}^{\left(r\right)}\right)$.(4): Calculate $\left|\tilde{Y}{\left[n\right]}^{\left(r+1\right)}-\tilde{Y}{\left[n\right]}^{\left(r\right)}\right|$. If $|\tilde{Y}{\left[n\right]}^{\left(r+1\right)}-\tilde{Y}{\left[n\right]}^{\left(r\right)}|<\epsilon_{2}$, set $\Lambda_{w}=\Lambda_{w}+10$ go to step (3), else set $\tilde{Y}{\left[n\right]}^{\left(r\right)}=\tilde{Y}{\left[n\right]}^{\left(r+1\right)}$,
r=r+1 and go to step (2).(5): update $Y{\left[n\right]}^{\left(r\right)}=\tilde{Y}{\left[n\right]}^{\left(r\right)}/t^{\left(r\right)}$ as the optimal solution of (P4).

### 3.4. Active IRS Optimization

For given user scheduling S, UAV trajectory Q, and beamforming vector W, the sub-problem of optimizing Ψ can be expressed as

(55a) $$\left(P5\right):\underset{\Psi }{max}R_{sec}=\frac{1}{N}\overset{N}{\underset{n=1}{\sum }}\left(\overset{J}{\underset{j=1}{\sum }}\alpha_{j}\left[n\right]R_{Bj}\left[n\right]-R_{E}\left[n\right]\right)$$

(55b) $$P_{IRS}\left[n\right]\leq P_{A},\forall n,$$

(55c) $$\left|\Psi \left[k,k\right]\right|\leq \eta_{k},\forall n,\forall k,$$

Provided that ψ[n] always appears in quadratic form, the SDR method is applied again as same as in (P4.2). Let $V\left[n\right]=\left[\begin{array}{c} \psi \left[n\right]\\ 1 \end{array}\right]\left[\begin{array}{cc} \psi {\left[n\right]}^{H} & 1 \end{array}\right]$, and the original problem can be rewritten as follows:

(56a) $$\left(P5.1\right):\underset{V}{max}\;C\left(V\right)=\frac{1}{N}\overset{N}{\underset{n=1}{\sum }}\left({\overset{-}{C}}_{Bj}\left[n\right]-{\overset{-}{C}}_{E}\left[n\right]\right)$$

(56b) $$tr\left(H_{U}\left[n\right]V\left[n\right]\right)\leq {\tilde{P}}_{A}\left[n\right],\forall n,$$

(56c) $$V\left[k,k\right]\leq \eta_{k}^{2},\forall n,\forall k,$$

(56d) V[K+1,K+1]=1,∀n,

(56e) V[n]≽0,∀n,

where

(57) $${\overset{-}{C}}_{j}\left[n\right]=\log_{2}\left(tr\left(H_{Uj}\left[n\right]V\left[n\right]\right)\right)-\log_{2}\left(tr\left(H_{Ij}\left[n\right]V\left[n\right]\right)\right),$$

(58) $$\tau_{j}=\sigma_{j}^{2}+h_{Uj}\left[n\right]w\left[n\right]w^{H}\left[n\right]h_{Uj}^{H}\left[n\right],$$

(59) $$H_{U}\left[n\right]=\left[\begin{array}{cc} diag\left(H_{UI}\left[n\right]w\left[n\right]\right)diag{\left(H_{UI}\left[n\right]w\left[n\right]\right)}^{H}+\sigma_{I}^{2}I & 0_{K\times 1}\\ 0_{1\times K} & 0 \end{array}\right],$$

(60) $$H_{Uj}\left[n\right]=\left[\begin{array}{cc} H_{j}\left[n\right]w\left[n\right]w^{H}\left[n\right]H_{j}^{H}\left[n\right]+{\bar{H}}_{j}\left[n\right] & H_{j}\left[n\right]w\left[n\right]w^{H}\left[n\right]h_{Uj}^{H}\left[n\right]\\ h_{Uj}\left[n\right]w\left[n\right]w^{H}\left[n\right]H_{j}^{H}\left[n\right] & \tau_{j} \end{array}\right],$$

(61) $$H_{Ij}=\left[\begin{array}{cc} {\bar{H}}_{j}\left[n\right] & 0_{n\times 1}\\ 0_{1\times n} & \sigma_{j}^{2} \end{array}\right],$$

(62) $$H_{j}\left[n\right]=diag\left(h_{Ij}\left[n\right]\right)H_{UI}\left[n\right],$$

(63) $${\bar{H}}_{j}\left[n\right]=\sigma_{I}^{2}diag\left(h_{Ij}\left[n\right]\right)diag{\left(h_{Ij}\left[n\right]\right)}^{H},j\in \left\{Bj,E\right\}.$$

Due to the objective function’s non-convexity, we can relax the problem and use the MM algorithm to iteratively optimize the relaxed one. The penalty-based strategy is used once more to recover the rank-1 solution throughout each iteration. At feasible point $\tilde{V}$, C(V) can be approximated by its first-order Taylor expansion:

$$C\left(V\right)\geq \log_{2}\left(tr\left(H_{UBj}\left[n\right]V\left[n\right]\right)\right)+\log_{2}\left(tr\left(H_{IE}\left[n\right]V\left[n\right]\right)\right)\;-\log_{2}\left(tr\left(H_{IBj}\left[n\right]\tilde{V}\left[n\right]\right)\right)-\log_{2}\left(tr\left(H_{UE}\left[n\right]\tilde{V}\left[n\right]\right)\right)\;-\log_{2}\left(tr\left(H_{IBj}\left[n\right]\tilde{V}\left[n\right]\right)\right)+\frac{1}{ln_{2}}tr\left(\frac{H_{IBj}\left[n\right]}{tr\left(H_{IBj}\left[n\right]\tilde{V}\left[n\right]\right)}\right)\left(V\left[n\right]-\tilde{V}\left[n\right]\right)$$

(64) $$-\ln\left(tr\left(H_{UE}\left[n\right]\tilde{V}\left[n\right]\right)\right)+\frac{1}{\ln_{2}}tr\left(\frac{H_{UE}\left[n\right]}{tr\left(H_{UE}\left[n\right]\tilde{V}\left[n\right]\right)}\right)\left(V\left[n\right]-\tilde{V}\left[n\right]\right)=\tilde{C}\left(V;\tilde{V}\right).$$

Here, $\tilde{C}\left(V;\tilde{V}\right)$ is a surrogate function, after dropping the constant term, the beamforming design problem can be recast into a convex form as

(65a) $$\left(P5.2\right):\underset{V}{max\;}\frac{1}{N}\overset{N}{\underset{n=1}{\sum }}\left(\log_{2}\left(tr\left(H_{UBj}\left[n\right]V\left[n\right]\right)\right)+\log_{2}\left(tr\left(H_{IE}\left[n\right]V\left[n\right]\right)\right)\right)\;-\frac{1}{N}\overset{N}{\underset{n=1}{\sum }}\left(\frac{1}{ln_{2}}tr\left(\left(\frac{H_{IBj}\left[n\right]}{tr\left(H_{IBj}\left[n\right]\tilde{V}\left[n\right]\right)}+\frac{H_{UE}\left[n\right]}{tr\left(H_{UE}\left[n\right]\tilde{V}\left[n\right]\right)}\right)V\left[n\right]\right)\right),$$

(65b) (56b), (56c),(56d),(56e).

(P5.2) can be directly optimized using the CVX solver, then the rank-1 solution is recovered by adding the penalty $\Lambda_{\Psi }\left[n\right]=tr(V\left[n\right])-u_{max}{\left(V{\left[n\right]}^{\left(r\right)}\right)}^{H}V\left[n\right]u_{max}\left(V{\left[n\right]}^{\left(r\right)}\right)$. After getting optimal V, the initial reflecting matrix can be rewritten as $\psi \left[n\right]=diag\left(\left(u_{max}\left(V\left[n\right]\right)\sqrt{\lambda_{\max}\left(V\left[n\right]\right)}\right)\left[1:K\right]\right)$. The phase coefficient and amplification coefficient can be expressed as follows:

(66) $$\Theta \left[n\right]=diag\left(\exp\left(j\;arg\left(\psi^{*}\left[n\right]\right)\right)\right),\forall n,$$

(67) $$p\left[n\right]=diag\left(\exp\left(-j\;arg\left(\psi^{*}\left[n\right]\right)\right)\right)\psi^{*}\left[n\right],\forall n.$$

Algorithm 2 demonstrates the algorithm’s rationale. The SDR algorithm is adopted to convert the original sub-problem into an SDP format. Applying the MM algorithm, a surrogate function is constructed to replace the objective function in (P5.1). By iteratively solving problem (P5.2) optimally, the convergence of C(V) promised. In each iteration, the penalty-based method is applied to recover the rank-1 solution.

**Algorithm 2** Active IRS Control Algorithm for Problem (P5).(1): Initialization: maximum threshold $\epsilon_{3}>0$, feasible point $\tilde{V}$, Calculate $C{\left(V\right)}^{\left(r\right)}$ given $\tilde{V}$ and number of iterations r = 0.(2): Optimize V in (P5.2), with given $\tilde{V}$.(3): Calculate rank(V). If rank(V)>1, the penalty-based method is applied to recover the rank-1 solution.(4): Calculate $C{\left(V\right)}^{\left(r+1\right)}$ given the rank-1 solution V.(5): Calculate $\left|C{\left(V\right)}^{\left(r+1\right)}-C{\left(V\right)}^{\left(r\right)}\right|$. If $|C{\left(V\right)}^{\left(r+1\right)}-C{\left(V\right)}^{\left(r\right)}|<\epsilon_{3}$, then the algorithm converges, go to step (6), else set r = r +1, $\tilde{V}=V$ and go to step (2).(6): Update $\psi \left[n\right]=diag\left(\left(u_{max}\left(V\left[n\right]\right)\sqrt{\lambda_{\max}\left(V\left[n\right]\right)}\right)\left[1:K\right]\right)$ as the optimal solution of (P5).

### 3.5. Overall Algorithm Description

The overall algorithm for solving the issue (P1) is outlined in Algorithm 3, where the BCD algorithm is applied, in accordance with the results gained in the previous four sub-problems. The original problem’s sub-optimal solution is found by solving the sub-problems in turn.

(P4.4) is an approximate solution to the sub-problem obtained from the decomposition of the original problem, and the convergence analysis of the BCD method cannot be applied directly. The convergence of Algorithms 1 and 2 are proved in Appendix A and Appendix B, respectively.

**Algorithm 3** Joint Optimization Algorithm for Maximizing Average Secrecy Capacity.(1): Initialization: maximum threshold $\epsilon_{4}>0$, feasible point $Q^{\left(0\right)},\;W^{\left(0\right)},\;\Psi^{\left(0\right)}$ and iteration r = 1.(2): Obtain $S^{\left(r\right)}$ with given $Q^{\left(r-1\right)},\;W^{\left(r-1\right)},\;\Psi^{\left(r-1\right)}$ by solving (P2).(3): Obtain $Q^{\left(r\right)}$ with given $S^{\left(r-1\right)},\;W^{\left(r-1\right)},\;\Psi^{\left(r-1\right)}$ by solving (P3.2).(4): Obtain $W^{\left(r\right)}$ with given $S^{\left(r-1\right)},\;Q^{\left(r\right)},\;\Psi^{\left(r-1\right)}$ by solving (P4.4).(5): Obtain $\Psi^{\left(r\right)}$ with given $S^{\left(r-1\right)},\;Q^{\left(r\right)},\;W^{\left(r\right)}$ by solving (P5.2).(6): With Given $S^{\left(r\right)},\;Q^{\left(r\right)},\;W^{\left(r\right)}\;\mathrm{and}\;\Psi^{\left(r\right)}$, update $R_{\sec}{}^{\left(r\right)}$ set r = r + 1.(7): Calculate the increment of the target value $\Delta =R_{\sec}{}^{\left(r\right)}-R_{\sec}{}^{\left(r-1\right)}$, if $\Delta <\epsilon_{4}$, the algorithm converges, go to step (8), else go to step (2).(8): Output $S^{*},\;Q^{*},\;W^{*},\;\Psi^{*}\;\mathrm{and}\;R_{\sec}{}^{*}$.

Denote g(S,Q,W,Ψ) as the objective function of (P1), then consider that $S^{\left(r\right)}$ is the solution of (P2), $Q^{\left(r\right)}$ is the solution of (P3.2), $W^{\left(r\right)}$ is the solution of (P4.4), and $\Psi^{\left(r\right)}$ is the solution of (P5.2). According to the above convergence analysis, we get the following inference:

(68) $$g\left(S^{\left(r\right)},Q^{\left(r\right)},W^{\left(r\right)},\Psi^{\left(r\right)}\right)\leq g\left(S^{\left(r+1\right)},Q^{\left(r+1\right)},W^{\left(r+1\right)},\Psi^{\left(r+1\right)}\right).$$

The target value of problem (P1) is non-decreasing after each iteration of Algorithm 3. Since the target value of problem (P1) is bounded by a finite value, Algorithm 3 must converge to a stable point.

The computational complexity of solving (P2) is expressed as O(N(J+1)), the computational complexity of using the SCA algorithm for (P3) is $O\left({\left(10N\right)}^{3.5}\right)$ [31], and the computational complexity of solving semidefinite programming problems for (P4) and (P5) is expressed as $O\left(NM^{3}\right)$ and $O\left(N{\left(K+1\right)}^{3}\right)$ [32], respectively. Thus, the complexity of the overall algorithm is $O\left(R_{ite}\left(N\left(J+1\right)+{\left(10N\right)}^{3.5}+NM^{3}+N{\left(K+1\right)}^{3}\right)\right)$, where $R_{ite}$ indicates the total number of iterations [33].

## 4. Analysis of Simulation Results

This section presents numerical findings to demonstrate the effectiveness of the suggested joint optimization scheme based on an active IRS-aided UAV system in improving the system’s average secrecy capacity. In this section, we present simulation results to show the performance of the proposed secure transmission scheme. Specifically, we assume that the ground area is 500×500 m, where the fixed height of the UAV is 50 m with a maximum speed $V_{\max}=15\;m/s$, the starting position is at the origin, and the flight endpoint of the UAV is ${\left(500,500\right)}^{T}\;m$. We consider a scenario where a M=4 antenna UAV and a K=16 element active IRS are employed to cooperatively serve the J=4 legitimate users, whose positions are ${\left(50,200\right)}^{T}\;m,\;{\left(250,100\right)}^{T}\;m,\;{\left(400,100\right)}^{T}\;m,$ and ${\left(450,400\right)}^{T}\;m$, respectively. The active IRS adopts a fully connected architecture, which means there are L=16 amplifiers. The antenna arrays of the UAV and the active IRS are assumed to be a uniform linear array and UPA, respectively, where the antenna spacings are both λ/2, λ is the frequency of the carrier wave. The position of the single passive eavesdropper is ${\left(500,200\right)}^{T}\;m$. There are also fixed active IRSs located at ${\left(350,150\right)}^{T}\;m$ with a height of 10 m. The UAV flies at a constant power $P_{fly}=29.7\;\mathrm{dBm}$, the hardware static power of the active IRS, which corresponds to the phase shift and amplifier are $P_{irs}=P_{amp}=10\;\mathrm{dBm}$, the maximum power budget at UAV and active IRS are $P_{U}=P_{A}=30\;\mathrm{dBm}$, δ=1 s is the time slot. Moreover, $\sigma_{I}^{2},\;\sigma_{B}^{2},\;\sigma_{E}^{2}=-110\;\mathrm{dBm}$ and α=2.2, β=2.5, κ=3, $\rho_{0}=-30\;\mathrm{dBm}$, are the power of AWGN, the path loss exponent, the Rician factor, and the reference channel gain, respectively. The default simulation parameters are shown in Table 3 unless otherwise specified.

### 4.1. Performance Simulation Results

**Figure 3:**
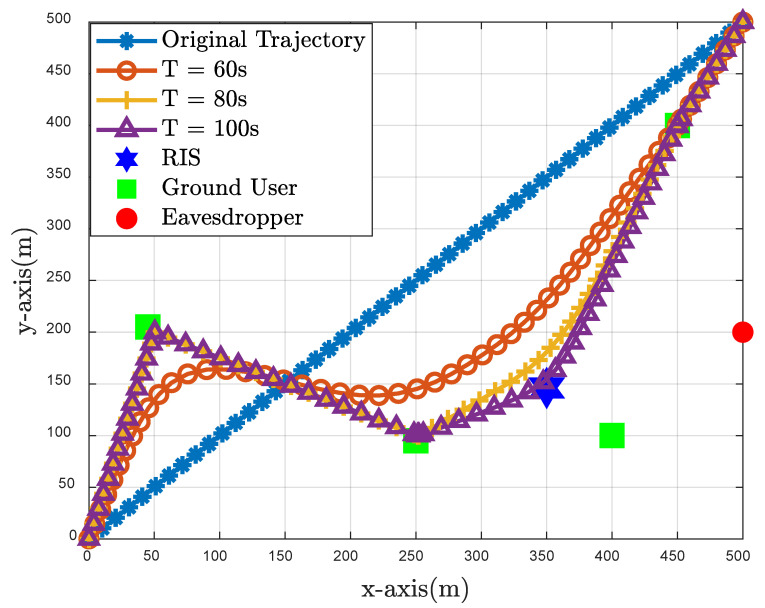
UAV trajectoies at different cycle times.

Figure 3 illustrates the distribution of four legitimate users, the eavesdropper as well as the active IRS, and plots the optimal trajectory of the UAV under different flight time conditions. Due to the “multiplicative fading” effect introduced by IRS, the UAV can balance the cascaded channel conditions by flying around the IRS and legitimate users and away from eavesdroppers, providing a safer quality of communication for legitimate users. Four situations are represented as four actual scenarios: for the original strategy, the UAV can only fly in an approximately straight path; when T=60 s, the UAV will try to fly to the best location to serve each legitimate user; when T=80 s, the UAV can reach each optimal location and stay appropriately; when T=100 s, the UAV will stay in the optimal location longer, providing a safer and better quality of service to legitimate users.

**Figure 4:**
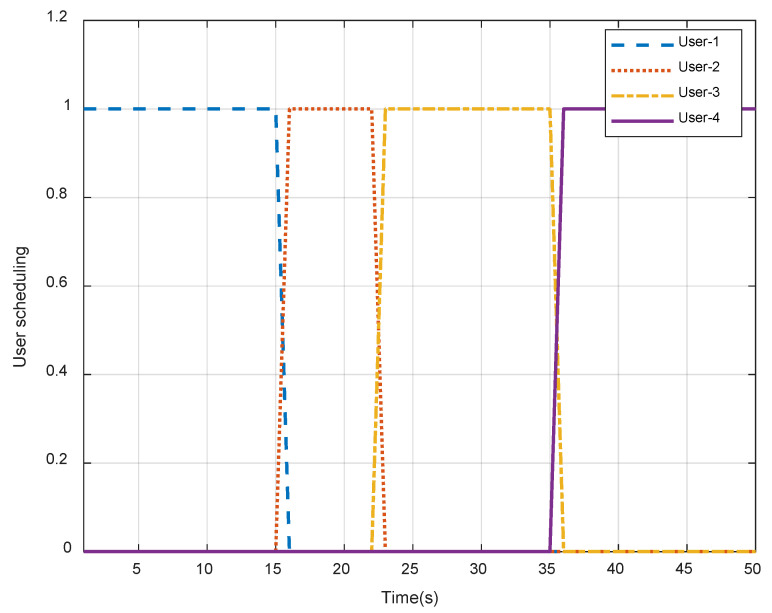
User scheduling in each time slot.

Figure 4 shows the time allocation and scheduling of four users in each time slot. In order to maximize the average secrecy capacity, the UAV should select appropriate users to transmit signals. It is shown that at the beginning, user-1 can obtain a higher secrecy rate and be selected. Until the 16th slot, user-2 can have a more secure communication environment due to the changes in UAV position, beamforming, and reflection parameters of the IRS, so user-2 becomes the served user. Once a user is selected for service, the UAV will send signals to that the user to obtain the maximum average secrecy capacity. In a multi-user scenario where time is sufficient, the UAV will first select the service user $Bj^{\left(k\right)}$ and fly to the optimal location. Taking the flight constraints into account, the UAV will stay for an appropriate amount of time and fly to the next optimal position, simultaneously sending a signal to $Bj^{\left(c+1\right)}$ as so on until it reaches the destination.

### 4.2. Performance Comparison of Different Optimization Algorithms

**Figure 5:**
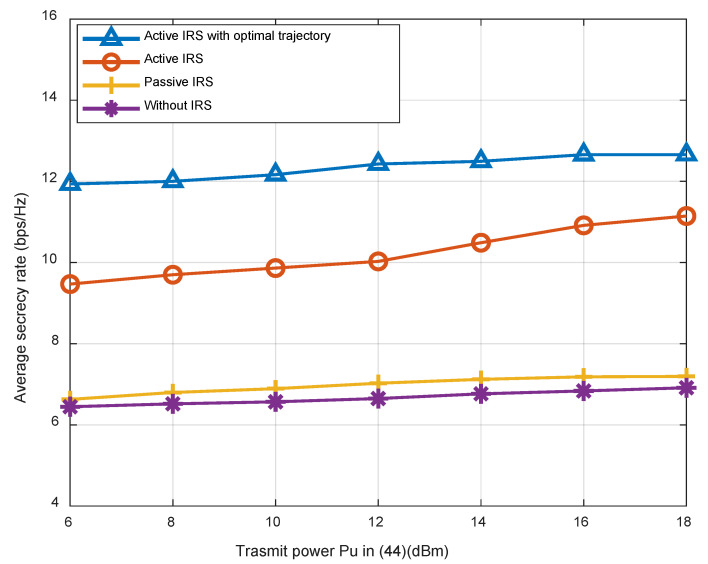
Average secrecy capacity of different schemes with respect to ${\tilde{P}}_{U}$.

Figure 5 shows a comparison between the suggested scheme and the other two benchmarks in terms of the average secrecy capacity with respect to various values of ${\tilde{P}}_{U}$ in (44), which is equivalent to the transmitting power. We considered a scenario where a M=4 antenna UAV and a K=16 element active IRS are employed cooperatively. In addition, it is worth noting that for the active scheme ${\tilde{P}}_{U}$ is used for transmitting antennas and the same power ${\tilde{P}}_{U}$ for reflecting signals, while for the passive schemes $2*{\tilde{P}}_{U}$ is used for transmitting signals. It is shown that the rate of all schemes will increase with the increase in ${\tilde{P}}_{U}$ to varying degrees. For example, at ${\tilde{P}}_{U}=18\;\mathrm{dBm}$, the active IRS achieves an improvement of 55.4% and 61.7% compared to the case with passive IRS and without IRS, respectively. By jointly optimizing user scheduling, UAV trajectory, beamforming, and the reflecting matrix, the proposed joint optimization strategy can outperform the benchmark schemes in terms of average secrecy capacity, proving that it is an efficient way to enhance security performance.

**Figure 6:**
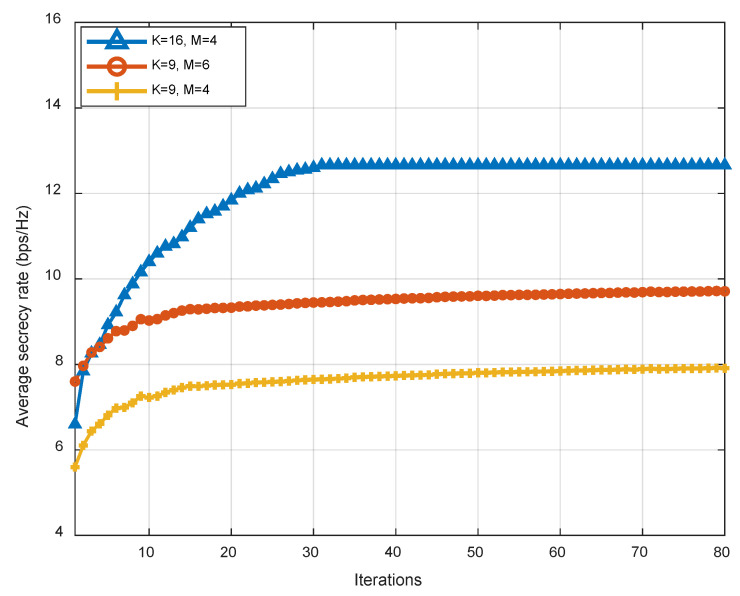
Convergence of Algorithm 3 under different settings of K and M.

Figure 6 shows the curve of the performance of the average security capacity with different settings of the number of IRS reflector units K and the number of antennas of UAV M. The transmit power ${\tilde{P}}_{U}=18\;\mathrm{dBm}$. As can be seen from the figure, our scheme can achieve acceptable performance in the above three settings. By comparing the curve of the (K=16, M=4) and (K=9, M=4) schemes, we can find that the average secrecy capacity will increase significantly with the increase in K, since more reflecting units can better align the signal to the served user. By comparing the curve of the (K=9,  M=6) and (K=9,  M=4) schemes, it is verified that the beamforming capability was also improved to some extent by increasing M, with a further improvement in secure communication performance.

**Figure 7:**
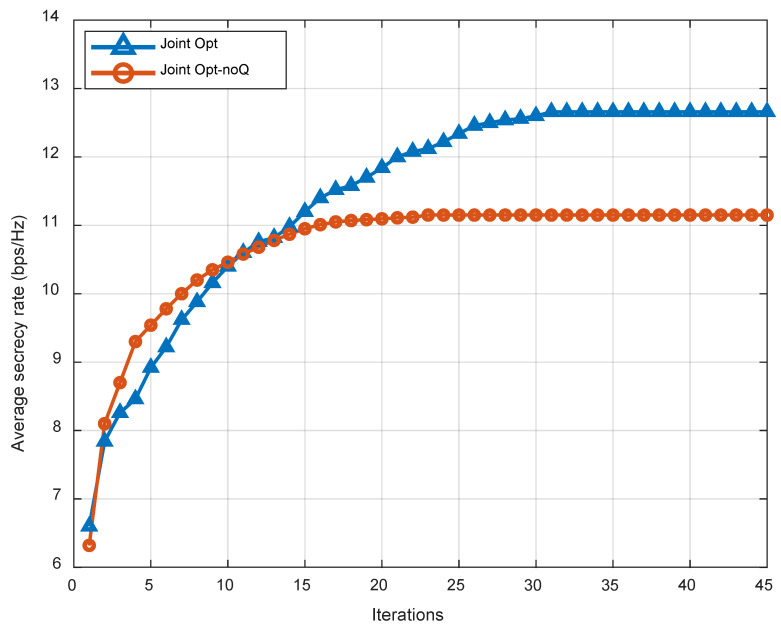
Convergence of different schemes.

The convergence of the proposed joint optimization algorithm (Algorithm 3) and the benchmark algorithm is depicted in Figure 7. The average secrecy capacity scheme under consideration is 11.9% higher than the benchmark without optimal trajectory. Additionally, the method converges after roughly 30 rounds.

.

## 5. Conclusions

This paper established a model for an active IRS-assisted UAV communication system where the UAV can dynamically select a user for service in each time slot based on its channel conditions. To maximize the average achievable secrecy capacity for the whole flight, the user scheduling, UAV trajectory, transmitting beamforming, and reflecting matrix are jointly optimized. However, the established problem was challenging to solve because of the objective non-convex function and tightly coupled variables. Therefore, an algorithm based on BCD is adopted to solve it. Initially, we decoupled the original issue into four sub-problems, and then the SCA technique, CCT method, and MM algorithm were applied to convert the original sub-problems into convex forms. Furthermore, we solved the above four sub-problems alternately based on the BCD algorithm and finally obtained the equivalent solution of the original problem. Numerical results show that the active IRS-assisted UAV communication scheme can efficiently weaken the effect of the “multiplicative fading” and significantly improved the secrecy capacity. In future research, we will consider the issues of IRS discrete designing and new advanced multi-access techniques for next-generation networks, such as NOMA and RSMA. Moreover, the DRL algorithm can be applied to solve the related optimization problems.

## Figures and Tables

**Table 1:** Summary of references [13,14,15,16,23,24].

Reference	Density, Type, and Mobility of UAV Transmitter	Communication Scenario	Type of IRS	Objective	Optimization Methods
[13]	Single UAV/Base Staion/Mobile	Ambient Backscatter Communication and /Unmodulated Backscatter Communication/Multiple users/Multiple Eves	Passive IRS	Maximizing the average secrecy rate	BCD + SDR + Q-learning
[14]	Single UAV/Relay/Fixed	UAV-Mounted IRS and Jammer/Fixed IRS/Single User/Multiple Eves	Passive IRS	Maximizing the minimum secrecy rate	BCD + SCA + SDR + DDPG
[15]	Single UAV/Base Staion/Mobile	Fixed IRS/Single User/Single Eve	Passive IRS	Maximizing the average secrecy rate	BCD + SCA
[16]	Single UAV/Base Staion/Mobile	Fixed IRS/Multiple Users/Multiple Eves	Passive IRS	Maximizing the minimum average secrecy rate	BCD + SCA
[23]	N/A	Fixed IRS/Single User/Single Eve	Active IRS	Maximizing the secrecy rate	LCAJP
[24]	Multiple UAVs/Base Station/Fixed	Multiple Fixed IRSs/Multiple Users	Hybrid IRS	Maximizing the minimum transmission rate	BCD + SCA

**Table 2:** Summary of symbols.

Symbol	Description
J	number of ground legitimate users
M	number of UAV antennas
K	number of the elements of active IRS
N	UAV flight duration
$H_{U}$	altitude of UAV
$H_{I}$	altitude of active IRS
q	horizontal coordinate of UAV
$w_{Bj}$	coordinate of legitimate users
$w_{E}$	coordinate of eavesdropper
$w_{I}$	horizontal coordinate of active IRS
$V_{\max}$	maximum flight speed of UAV
δ	time slot
$\rho_{0}$	channel power gain
α, β	path loss exponent
$\sigma_{I}^{2},\sigma_{B}^{2},\sigma_{E}^{2}$	noise power
$P_{fly}$	dissipated power consumed at UAV
$P_{irs}$	static power of active IRS corresponding to phase-shift circuit
$P_{amp}$	static power of active IRS corresponding to amplifier circuit
$P_{U}$	maximum power of UAV
$P_{A}$	maximum power of the active IRS
ζ	reciprocal of energy conversion coefficient at the transmitter of UAV
ξ	reciprocal of energy conversion coefficient at the active IRS
d	antenna separation at UAV
$d_{0}$	separation of elements at IRS

**Table 3:** Simulation parameters.

**Symbol**	**Value**	**Symbol**	**Value**
J	4	$V_{\max}$	15 m/s
M	4	δ	1s
K	16	$\rho_{0}$	−30 dBm
L	16	α, β	2.2, 2.5
N	50	$\sigma_{I}^{2},\sigma_{B}^{2},\sigma_{E}^{2}$	−110 dBm
$H_{U}$	50 m	$P_{fly}$	29.7 dBm
$H_{I}$	10 m	$P_{irs}$	−20 dBm
$\;q_{0}$	${\left(0,\;0\right)}^{T}\;m$	$P_{amp}$	−20 dBm
$q_{F}$	${\left(500,\;500\right)}^{T}\;m$	$P_{U}$	30 dBm
$w_{Bj}$	${\left(50,200\right)}^{T\;}m,\;{\left(250,100\right)}^{T}\;m,\;{\left(400,100\right)}^{T}\;m,\;{\left(450,400\right)}^{T}\;m$		
$w_{E}$	${\left(500,\;200\right)}^{T}\;m$	$P_{A}$	18 dBm
$w_{I}$	${\left(350,\;150\right)}^{T}\;m$	ζ, ξ	1.1
κ	3	$d,\;d_{0}$	λ/2

## Data Availability

The data presented in this study are available in Table 3.

## Abbreviations

The following abbreviations are used in this manuscript:

IRS	intelligent reflecting surface
UAV	unmanned aerial vehicles
BCD	block coordinate descent
LoS	line of sight
NLoS	non-line of sight
MISO	multiple input single output
MIMO	multiple input multiple output
SCA	successive convex approximation
SDR	semidefinite relaxation
CCT	Charnes–Cooper transformation
MM	majorization-minimization
ULA	uniform linear array
UPA	uniform plane array
AoA	angle of arrival
AoD	angle of departure

## Appendix A

The equivalent form of the problem (P4.4) is expressed as

$$\underset{\tilde{Y},t}{min}\;\;\frac{1}{N}\overset{N}{\underset{n=1}{\sum }}\left(-\left(t\left[n\right]+{\tilde{h}}_{B}\left[n\right]\tilde{Y}\left[n\right]{\tilde{h}}_{B}^{H}\left[n\right]\right)\right)\;+\frac{1}{N}\overset{N}{\underset{n=1}{\sum }}\left(\Lambda_{w}\left[n\right]\left(tr\left(\tilde{Y}\left[n\right]p\left[n\right]-\lambda_{max}\left(\tilde{Y}{\left[n\right]}^{\left(r\right)}\right)\right)\right)\right)$$

(A1) $$-\frac{1}{N}\overset{N}{\underset{n=1}{\sum }}\left(\Lambda_{w}\left[n\right]\left(u_{max}{\left(\tilde{Y}{\left[n\right]}^{\left(r\right)}\right)}^{H}\left(\tilde{Y}\left[n\right]-\tilde{Y}{\left[n\right]}^{\left(r\right)}\right)u_{max}\left(\tilde{Y}{\left[n\right]}^{\left(r\right)}\right))\right)\right).$$

Denote $f\left(t,\tilde{Y}\right)$ as the objective function of (P4.4) and $\left(t{\left[n\right]}^{\left(r\right)},\tilde{Y}{\left[n\right]}^{\left(r\right)}\right)$ is a feasible solution to the aforementioned problem with a given $\Lambda_{w}\left[n\right]$. $\left(t{\left[n\right]}^{\left(r+1\right)},\tilde{Y}{\left[n\right]}^{\left(r+1\right)}\right)$ represents the optimal solution of (64), and one obtains that

$$-\left(t{\left[n\right]}^{\left(r+1\right)}+{\tilde{h}}_{B}\left[n\right]\tilde{Y}{\left[n\right]}^{\left(r+1\right)}{\tilde{h}}_{B}^{H}\left[n\right]\right)+\Lambda_{w}\left[n\right](tr\left(\tilde{Y}{\left[n\right]}^{\left(r+1\right)}\right)-\lambda_{max}\left(\tilde{Y}{\left[n\right]}^{\left(r\right)}\right)\;-u_{max}{\left(\tilde{Y}{\left[n\right]}^{\left(r\right)}\right)}^{H}\left(\tilde{Y}{\left[n\right]}^{\left(r+1\right)}-\tilde{Y}{\left[n\right]}^{\left(r\right)}\right)u_{max}\left(\tilde{Y}{\left[n\right]}^{\left(r\right)}\right))$$

(A2) $$\leq -\left(t{\left[n\right]}^{\left(r\right)}+{\tilde{h}}_{B}\left[n\right]\tilde{Y}{\left[n\right]}^{\left(r\right)}{\tilde{h}}_{B}^{H}\left[n\right]\right)+\Lambda_{w}\left[n\right]\left(tr\left(\tilde{Y}{\left[n\right]}^{\left(r\right)}\right)-\lambda_{max}\left(\tilde{Y}{\left[n\right]}^{\left(r\right)}\right)\right).$$

Moreover, the sub-gradient of $\lambda \left(\tilde{Y}{\left[n\right]}^{\left(r\right)}\right)$ satisfies

(A3) $$\partial \lambda \left(\tilde{Y}{\left[n\right]}^{\left(r\right)}\right)=u_{max}\left(\tilde{Y}{\left[n\right]}^{\left(r\right)}\right)u_{max}{\left(\tilde{Y}{\left[n\right]}^{\left(r\right)}\right)}^{H},$$

(A4) $$\forall Y\geq 0,\;\lambda_{max}\left(Y\right)-\lambda_{max}\left(X\right)\geq u_{max}{\left(\tilde{Y}{\left[n\right]}^{\left(r\right)}\right)}^{H}\left(Y-X\right)u_{max}\left(\tilde{Y}{\left[n\right]}^{\left(r\right)}\right).$$

Thus, we obtain the following

$$f\left(t{\left[n\right]}^{\left(r+1\right)},\tilde{Y}{\left[n\right]}^{\left(r+1\right)}\right)=-\left(t{\left[n\right]}^{\left(r+1\right)}+{\tilde{h}}_{B}\left[n\right]\tilde{Y}{\left[n\right]}^{\left(r+1\right)}{\tilde{h}}_{B}^{H}\left[n\right]\right)\;+\Lambda_{w}\left[n\right](tr\left(\tilde{Y}{\left[n\right]}^{\left(r+1\right)}\right)-\lambda_{max}\left(\tilde{Y}{\left[n\right]}^{\left(r+1\right)}\right)-\lambda_{max}\left(\tilde{Y}{\left[n\right]}^{\left(r\right)}\right)+\lambda_{max}\left(\tilde{Y}{\left[n\right]}^{\left(r\right)}\right))\;\leq -\left(t{\left[n\right]}^{\left(r+1\right)}+{\tilde{h}}_{B}\left[n\right]\tilde{Y}{\left[n\right]}^{\left(r+1\right)}{\tilde{h}}_{B}^{H}\left[n\right]\right)+\Lambda_{w}\left[n\right]\left(tr\left(\tilde{Y}{\left[n\right]}^{\left(r+1\right)}\right)-\lambda_{max}\left(\tilde{Y}{\left[n\right]}^{\left(r\right)}\right)\right)\;-\Lambda_{w}\left[n\right]\left(u_{max}{\left(\tilde{Y}{\left[n\right]}^{\left(r\right)}\right)}^{H}\left(\tilde{Y}{\left[n\right]}^{\left(r+1\right)}-\tilde{Y}{\left[n\right]}^{\left(r\right)}\right)u_{max}\left(\tilde{Y}{\left[n\right]}^{\left(r\right)}\right)\right)\;\leq -\left(t{\left[n\right]}^{\left(r\right)}+{\tilde{h}}_{B}\left[n\right]{\tilde{Y}}^{\left(r\right)}\left[n\right]{\tilde{h}}_{B}^{H}\right)+\Lambda_{w}\left[n\right]\left(tr\left(\tilde{Y}{\left[n\right]}^{\left(r\right)}\right)-\lambda_{max}\left(\tilde{Y}{\left[n\right]}^{\left(r\right)}\right)\right)$$

(A5) $$=f\left(t{\left[n\right]}^{\left(r\right)},\tilde{Y}{\left[n\right]}^{\left(r\right)}\right).$$

For a given $\Lambda_{w}\left[n\right]$, $f\left(t{\left[n\right]}^{\left(r\right)},\tilde{Y}{\left[n\right]}^{\left(r\right)}\right)\leq f\left(t{\left[n\right]}^{\left(r+1\right)},\tilde{Y}{\left[n\right]}^{\left(r+1\right)}\right)$ holds. Therefore, $f\left(t{\left[n\right]}^{\left(r\right)},\tilde{Y}{\left[n\right]}^{\left(r\right)}\right)$ is non-increasing, which guarantees the convergence of Algorithm 1.

## Appendix B

For any concave function, we have $f\left(x\right)\leq f\left(\tilde{x}\right)+{\left(\nabla f\left(\tilde{x}\right)\right)}^{T}\left(x-\tilde{x}\right)$. $tr\left(H_{IBj}\left[n\right]V\left[n\right]\right)$ and $tr\left(H_{UE}\left[n\right]V\left[n\right]\right)$ are linear functions, $\log_{2}\left(tr\left(H_{IBj\left[n\right]}V\left[n\right]\right)\right)$ and $\log_{2}\left(tr\left(H_{UE}\left[n\right]V\left[n\right]\right)\right)$ are concave functions, then the first-order Taylor expansion of $\log_{2}\left(tr\left(H_{IBj}\left[n\right]V\left[n\right]\right)\right)$ and $\log_{2}\left(tr\left(H_{UE}\left[n\right]V\left[n\right]\right)\right)$ with a given feasible $\tilde{V}$ can be expressed as

(A6) $$\log_{2}\left(tr\left(H_{IBj}\left[n\right]V\left[n\right]\right)\right)\;\leq \log_{2}\left(tr\left(H_{IBj}\left[n\right]\tilde{V}\left[n\right]\right)\right)+\frac{1}{ln_{2}}tr\left(\frac{H_{IBj}\left[n\right]}{tr\left(H_{IBj}\left[n\right]\tilde{V}\left[n\right]\right)}\right)\left(V\left[n\right]-\tilde{V}\left[n\right]\right),$$

(A7) $$\log_{2}\left(tr\left(H_{UE}\left[n\right]V\left[n\right]\right)\right)\;\leq \log_{2}\left(tr\left(H_{UE}\left[n\right]\tilde{V}\left[n\right]\right)\right)+\frac{1}{ln_{2}}tr\left(\frac{H_{UE}\left[n\right]}{tr\left(H_{UE}\left[n\right]\tilde{V}\left[n\right]\right)}\right)\left(V\left[n\right]-\tilde{V}\left[n\right]\right).$$

Bringing them to C(V), then we have

$$C\left(V\right)=\log_{2}\left(tr\left(H_{UBj}\left[n\right]V\left[n\right]\right)\right)+\log_{2}\left(tr\left(H_{IE}\left[n\right]V\left[n\right]\right)\right)\;-\log_{2}\left(tr\left(H_{UE}\left[n\right]V\left[n\right]\right)\right)-\log_{2}\left(tr\left(H_{IBj}\left[n\right]V\left[n\right]\right)\right)\;\geq \log_{2}\left(tr\left(H_{UBj}\left[n\right]V\left[n\right]\right)\right)+\log_{2}\left(tr\left(H_{IE}\left[n\right]V\left[n\right]\right)\right)\;-\log_{2}\left(tr\left(H_{IBj}\left[n\right]\tilde{V}\left[n\right]\right)\right)-\log_{2}\left(tr\left(H_{UE}\left[n\right]\tilde{V}\left[n\right]\right)\right)\;-\log_{2}\left(tr\left(H_{IBj}\left[n\right]\tilde{V}\left[n\right]\right)\right)+\frac{1}{ln_{2}}tr\left(\frac{H_{IBj}\left[n\right]}{tr\left(H_{IBj}\left[n\right]\tilde{V}\left[n\right]\right)}\right)\left(V\left[n\right]-\tilde{V}\left[n\right]\right)\;-\log_{2}\left(tr\left(H_{UE}\left[n\right]\tilde{V}\left[n\right]\right)\right)+\frac{1}{\ln_{2}}tr\left(\frac{H_{UE}\left[n\right]}{tr\left(H_{UE}\left[n\right]\tilde{V}\left[n\right]\right)}\right)\left(V\left[n\right]-\tilde{V}\left[n\right]\right)$$

(A8) $$=\tilde{C}\left(V;\tilde{V}\right),$$

where $\tilde{C}\left(V;\tilde{V}\right)$ is a surrogate function since four key conditions hold [34]: ①$C\left(V\right)\geq \tilde{C}\left(V;\tilde{V}\right)$; ②$C\left(\tilde{V}\right)=\tilde{C}\left(V;\tilde{V}\right)$; ③$\nabla \tilde{C}\left(V;\tilde{V}\right){|}_{V=\tilde{V}}=\nabla C\left(V\right){|}_{V=\tilde{V}}$; ④$\tilde{C}\left(V;\tilde{V}\right)$ and are continuous in V and $\tilde{V}$. According to the key property of the MM algorithm, the convergence of Algorithm 2 is guaranteed.
